# Advances in assessing *Sabellaria spinulosa* reefs for ongoing monitoring

**DOI:** 10.1002/ece3.4292

**Published:** 2018-07-12

**Authors:** Christopher Jenkins, Jacqueline Eggleton, Jon Barry, Joey O'Connor

**Affiliations:** ^1^ Centre for Environment, Fisheries and Aquaculture Science (Cefas) Lowestoft UK; ^2^ Joint Nature Conservation Committee (JNCC) Peterborough UK

**Keywords:** biogenic reef, habitat mapping, Marine Protected Areas, monitoring, *Sabellaria spinulosa*

## Abstract

Standardized and repeatable data acquisition and analyses are required to enable the mapping and condition monitoring of reefs within Marine Protected Areas (MPAs). Changes in habitat condition must be reliably identified and reported to best support evidence‐based management. Biogenic reefs in temperate waters, that is, hard matter created by living organisms and raised above the seabed, provide food and shelter for many plant and animal species. This article explores the feasibility of habitat mapping, using remote sensing datasets, as well as metrics for repeatable and suitable assessment of areas of *Sabellaria spinulosa* for their status as biogenic reef. Data were gathered within the North Norfolk Sandbanks and Saturn Reef candidate Special Area of Conservation/Site of Community Importance in the southern North Sea. Six study areas were identified as potential locations of biogenic reef using previously acquired data, and these were targeted for further investigation using a combination of high resolution multibeam echosounder and sidescan sonar. Where potential *S. spinulosa* was identified from the acoustic data, a drop‐down camera system was employed for visual verification. Areas of known and potential *S. spinulosa* reef were mapped successfully at two of the six study areas, although future approaches should take careful consideration of the seabed morphology and predominant habitat backdrop to successfully interpret such data. Camera tows from *S. spinulosa* reef areas were broken up into 5‐s segments, with each segment scored for (a) average tube elevation; (b) average percentage cover; and (c) for the presence or absence of *S. spinulosa*. These metrics were utilized to create summary statistics, including a value of patchiness derived from presence/absence data, that is recommended for application as part of future monitoring programs. The application of this methodology could benefit wider assessments of similar threated or declining habitats such as intertidal *Mytilus edulis* beds on mixed and sandy sediments, Maerl beds, *Modioulus modiolus* beds, *Ostrea edulis* beds, and *Zostera* beds where patchiness may also be considered of environmental importance.

## 1 INTRODUCTION

The Ross Worm *Sabellaria spinulosa* is widely distributed in temperate waters, occurring as individuals but also forming reefs comprising many individuals on sandy and mixed/coarse sediments (Gubbay, 2007). High densities of *S. spinulosa* have been found to occur in the UK in the vicinity of the Wash and along the South Coast of the UK (Hendrick, 2007; Hendrick, Foster‐Smith, & Davies, 2011). Other reports of dense aggregations include records from the Bristol Channel (George & Warwick, 1985), the Dorset coast (Collins, 2003), the Thames Estuary (Attrill, Ramsay, Thomas, & Trett, 1996), the Northumberland coast (Jones, 1972) and the southern North Sea (BBL Company, 2006; BMT Cordah Ltd., 2003). In Scotland, dense *S. spinulosa* aggregations have been reported at Hilbre Island at the mouth of the Dee, from East Rocks, St Andrews (McIntosh, 1922) and to the south of Rattray Head on the north east coast (Braithwaite, Robinson, & Jones, 2006). Occurrences of other aggregations have been reported from north and west Wales (Hiscock, 1984) and Dublin Bay in Ireland (Walker & Rees, 1980). Given their potential ephemeral nature, such reefs may, however, not currently be actively forming new biogenic reef structures (OSPAR, 2013).

Annex I reefs have been defined by the revised EU Interpretation Manual (EC, 2007) as:

Reefs can be either biogenic concretions or of geogenic origin. They are hard compact substrata on solid and soft bottoms, which arise from the sea floor in the sublittoral and littoral zone. Reefs may support a zonation of benthic communities of algae and animal species as well as concretions and corallogenic concretions.

OSPAR^1^ specifically defines *S. spinulosa* reef in mixed substrata habitats as “comprised variously of sand, gravel, pebble and cobble, the Sabellaria covers 30% or more of the substrata and needs to be sufficiently thick and persistent to support an associated epibiota community which is distinct from surrounding habitats.”

*Sabellaria spinulosa* formed reef structures have been identified as being of conservation importance and are therefore afforded protection within the UK's network of Marine Protected Areas (MPAs), qualifying as Annex I habitat according to the European Commission (CEC, 2007). In order to function as an ecologically coherent network, MPA designation must be underpinned by a robust evidence base which effectively validates the presence and distribution of the protected features of conservation importance across the sites. It is equally important that decisions about the management approach implemented, to ensure adequate protection of biogenic reef within an MPA, is similarly underpinned by robust evidence regarding its distribution, spatial extent, and condition. *S. spinulosa* reefs are sensitive to a number of pressures, such as abrasion (OSPAR, 2010, Foster‐Smith & Hendrick, 2003; Gibb, Tillin, Pearce, & Tyler‐Walters, 2014; Holt, Rees, Hawkins, & Seed, 1998; Jones, Hiscock, & Connor, 2000), but may also be naturally ephemeral (Hendrick & Foster‐Smith, 2006). This adds a layer of complexity when making assessments of reef condition, in relation to the potential effects of anthropogenic activity, in the context of natural variability.

Methods to determine the presence, extent, and condition of *S. spinulosa* reef habitats commonly involve a combination of acoustic, for example, acoustic ground‐discrimination systems (e.g., Roxann), sidescan sonar (SSS), and/or multibeam echosounder (MBES) and groundtruthing, for example, video imagery collected via remotely operated vehicles (ROV) or drop‐down video systems (DDV), and/or physical samples collected by grab and/or beam trawl. These survey techniques have recently been explored by Limpenny et al. (2010). In particular, this study comprised an in‐depth analysis of the benefits and limitations of a variety of survey techniques which explored the identification and assessment of spatial extent of *S. spinulosa* reefs using a number of remote sensing approaches. Sidescan sonar was considered to be the most suitable tool, when used in combination with ground‐truthing data, for making assessments of potential areas of reef. Although bathymetric data collected using multibeam echosounders (MBES) were considered to be a useful complimentary approach, it was found to be less reliable at identifying the spatial extent of biogenic reef structures.

Pearce et al. (2014) explored approaches for mapping the spatial extent of *S. spinulosa* reef at the Thanet offshore windfarm site. This study presented a multi‐year mapping approach to assess potential impacts associated with an offshore infrastructure development. The assessment utilized sidescan sonar, along with MBES bathymetry and backscatter and ground‐truthing techniques, to explore temporal variability in reef distribution and spatial extent. The authors found a positive correlation between the windfarm development and an increase in reef extent. However, limitations such as the variability in personnel available to produce the habitat maps, on which the estimates of reef extent were based are noted. This limitation results in reduced confidence in the mapping outputs which had indicated an apparent temporal change in habitat extent. These outcomes were supported by the studies of both Coggan, Mitchell, White, and Golding (2007) and Diesing et al. (2014) who also emphasize the importance of repeatable methodologies for data acquisition and habitat mapping which also adopt an element of automation, thereby rendering the mapping outputs less influenced by the issues of subjectivity which arise as a result of manual interpretation.

**Figure 1:**
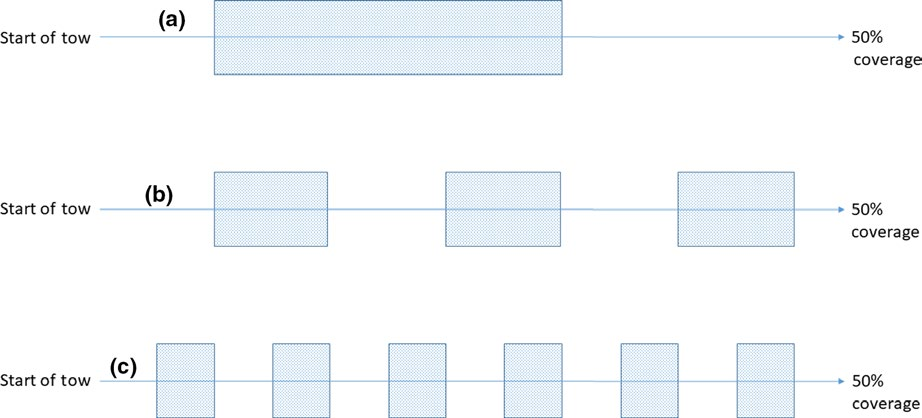
Schematic diagram representing three independent camera tows over patches of reef, all presenting 50% coverage (Top—a, a highly consolidated reef; Middle—b, a moderately consolidated reef; and Bottom—c, a poorly consolidated reef)

Reef structure and condition are commonly assessed by quantitatively measuring reef elevation from the seabed and patchiness (determined as percentage cover) within a defined area (Gubbay, 2007; Hendrick & Foster‐Smith, 2006). Video techniques are preferred over physical sampling as they are less destructive (Davies et al., 2001; Foster‐Smith & Hendrick, 2003), although physical sampling does provide an additional layer of information, for example, *S. spinulosa* densities and associated biodiversity (Pearce et al., 2011, 2014). Videos are initially segmented into broad habitat types (i.e., reef/no reef) based on major changes in the substratum (Coggan et al., 2007; Rees, 2009; Turner et al., 2016), with any change measuring less than 5 m^2^ considered as incidental patches (Davies et al., 2001). An estimate of tube height and percentage cover of reef is then determined for each segment containing reef. Using percentage cover as a proxy for patchiness as suggested by Gubbay (2007) is, however, likely to result in a measure of reef density rather than a measure of “true patchiness.” Patches could refer both to localized aggregations of reef interspersed by sediment or larger aggregations spread across a wider area, with localized patchiness within (Foster‐Smith & Hendrick, 2003). Reef areas may, therefore, demonstrate varying levels of patchiness, while still having similar values for percentage cover (Figure 1).

Foster‐Smith and Hendrick (2003) attempted to quantify the variability in reef patchiness at an aggregate extraction site, Area 107, in the Wash, UK, based on measurements of similarity between grab samples at different spatial separations. However, this method used information on differences in faunal assemblages rather than reef structure per se. Pearce et al. (2014) estimated *S. spinulosa* tube density, % cover and fauna from 3 to 5 replicate images per habitat, whilst Sheehan et al. (2010) used HD video to enable image capture of for quantitative analysis of rocky reef habitat. While these methods again provide quantitative information for statistical analysis of community structure, they are unlikely to provide sufficient information for quantitatively comparing the fine‐scale patchiness of the reef itself. Spatial patchiness of *S. spinulosa* reefs varies considerably in nature; therefore, assessing *S. spinulosa* reef following these methodologies may not enable an accurate measure of “true patchiness,” and hence condition, of reefs. Mortensen and Buhl‐Mortensen (2004) presented a methodology for quantitatively assessing fine‐scale distribution of deep water corals and habitat features. Videos were generally segmented at 30‐s intervals, but made shorter when abrupt changes in habitat occurred earlier. Patchiness of the coral distribution along the video transects was based on the presence/absence of coral species and determined using Morisita's index of dispersion (*I*_d_—Morisita, 1962). This index is a measure of how similar or different two sets of data are and ranges from 0 (no similarity) to 1 (complete similarity). However, this index has been criticized as it can give values larger than 1 which may lead to misleading interpretations (Chao, Chazdon, Colwell, & Shen, 2006). Various other statistics have been used to define patchiness of spatial data including Clark and Evans (1954) and the *G* statistic of Brown and Rothery (1978) (see also Dare & Barry, 1990). These statistical methods are based on nearest neighbor distances between points and are therefore not capable of distinguishing between different degrees of patchiness. Quantitatively comparing patchiness of reef between subsections of a video tow or among tows is therefore dependent both upon comparable coverage; that is, either the time of the tow/subsection and/or the distance covered (White, Mitchell, Coggan, Southern, & Golding, 2007) and readily understandable statistical measures of patchiness.

Consistent and repeatable methods of data acquisition, analysis, and assessment are essential for effective monitoring and management of biogenic reef structures. To that end, we use seabed survey data collected in the southern North Sea in 2013 to (a) critically evaluate data acquisition techniques for informing reef assessment (specifically seabed habitat mapping using remote sensing datasets and condition assessments using seabed imagery data), and their limitations; and (b) to propose further metrics and repeatable methods for reef assessment. While this work specifically considers *S. spinulosa* reefs, the conclusions drawn from this study are relevant to other elevated, biogenic reef forming, structures (such as *Modiolus* reefs) in the marine environment.

## 2 MATERIALS AND METHODS

### 2.1 Study site

**Figure 2:**
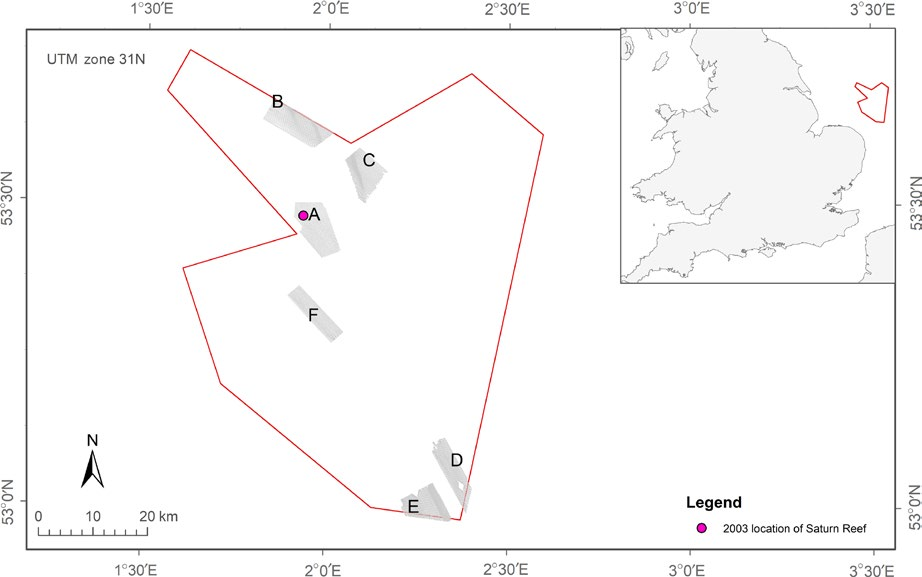
Location of the North Norfolk Sandbanks and Saturn Reef Site of Community Importance (inset) with study areas (a–f) of high resolution sidescan sonar and multibeam echosounder surveys (main map)

Our study site was located at the North Norfolk Sandbanks (NNSB) and Saturn Reef (SR) candidate Special Area of Conservation (cSAC)/Site of Community Interest (SCI) in the southern North Sea (JNCC, 2010), extending from approximately 40 km off the north east coast of Norfolk (Figure 2). In addition to areas of *S. spinulosa* reef, the cSAC/SCI encompasses a series of ten sandbank features (Leman, Inner, Ower, Well, Broken, Swarte, and four sandbanks collectively known as the Indefatigables), and associated fragmented smaller banks, which collectively represent the most extensive example of the offshore linear ridge sandbank feature in UK waters (Graham, Campbelle, Cavill, Gillespie, & Williams, 2001).

Conservation objectives for the cSAC/SCI are to restore the Annex I Sandbanks and Reef features (including *S. spinulosa* reef) to favorable condition such that the natural environmental quality, natural environmental processes, and extent are maintained and that the physical structure, diversity, community structure, and typical species representative of the Annex I habitats are restored (JNCC, 2012).

In 2003, the Saturn Reef *S. spinulosa* biogenic reef was identified within NNSB and SR cSAC/SCI, located between Swarte Bank and Broken Bank, and was determined to be an excellent example of the Annex I biogenic reef habitat (BMT Cordah, 2003). The reef was found to consist of thousands of consolidated sand tubes, made by *S. spinulosa*, creating a solid structure rising above the seabed. The spatial extent of Saturn Reef was estimated to cover an area of 0.375 km^2^, with a “core” area (0.125 km^2^) of near continuous (90% coverage), and high elevation (>10 cm high) reef. Areas of patchy reef (representing <10–50% coverage) were also observed which contained various shaped holes or comprised elongated strips, raised above surrounding seabed. Surrounding sediments included both tube debris and areas where *S. spinulosa* tubes were absent (e.g., silty sand/stones). Damage to the physical structure of the reef, which may have been the result of bottom trawling, was also observed, particularly in the south western part of the study area.

In 2006, during a subsequent survey of the Saturn Reef area (Limpenny et al., 2010), the previously observed biogenic reef structures were not found in the same location. It has not been determined as to whether this apparent absence was as a result of anthropogenic damage to the reef structures (e.g., by bottom trawling) or due to the possible ephemeral nature of this feature (OSPAR, 2013). However, the formation of a substantial reef of *S. spinulosa* in this area in 2003 does indicate favorable conditions for continued reef formation.

### 2.2 Data collection

Six study areas (A–F; Figure 2) within the NNSB and SR SCI were targeted for detailed investigation. Selection of these sites was based on a habitat suitability assessment, using expert judgment, where suitability was based on whether predominant habitat type and prevailing environmental conditions were considered potentially suitable for *S. spinulosa* recruitment and biogenic reef formation (Jenkins et al., 2015).

Acoustic survey lines were acquired at 200‐m line spacing to achieve 100% seafloor coverage using a high resolution sidescan sonar system (Edgetech FS‐4200 dual frequency 300/600 kHz). Simultaneous collection of multibeam echosounder data (Kongsberg EM2040 system operated at 200 kHz and deployed on the drop keel of the research vessel) was also collected, recognizing that full seafloor coverage would not be achieved with this technique at this line spacing within the water depths encountered.

Following an on‐board review of acoustic data, areas identified as having potential *S. spinulosa* reef signatures were targeted for the subsequent validation via the collection of video and stills data using a Kongsberg 14‐208, 5 megapixel, camera mounted in a rectangular drop frame. High‐power LED strip lights and a four‐point laser system with lasers set 17 cm apart (to provide scale) were also mounted to the frame. Video tows were a minimum of 10 min long. Still images were captured at regular 1‐min intervals and, in addition, opportunistically if specific features of interest were encountered.

### 2.3 Data processing

#### 2.3.1 Video analysis

A total of 152 videos were initially analyzed using Cefas video and stills processing protocol^2^ (Jenkins et al., 2015) whereby a change in substrate/habitat is recorded if it continues for more than 1 min. This method enables broad‐scale changes in substratum to be recorded; however, small changes in habitat are not recorded, but are noted as incidental patches (see also Davies et al., 2001; Turner et al., 2016).

Videos in which *S. spinulosa* reef was observed were analyzed further using a standardized method for determining reef patchiness developed by JNCC and Cefas (Jenkins et al., 2015). Fifty‐seven videos were split into 5‐s segments using an automated script in VLC Video Player, resulting in 3,283 five‐second segments. Data quality, presence/absence of *S. spinulosa* reef, percentage cover of reef, and an estimate of tube height were recorded for each 5‐s segment.

To account for the variations in the camera's field of view along a given video segment, percentage cover was estimated over the segment and divided by the number of times a new area of seabed was observed. Average elevation (i.e., the height of the reef from its base) was assessed using the four‐point laser system mounted on the drop frame which provided an accurate and consistent scale for measurement.

Measurements of percentage cover and elevation, as proposed by Gubbay (2007), for each 5‐s video segment were assigned a score relating to “reef status” using the modified reef structure matrix in Table 1, which illustrates the spatial variability of reef composition along each transect.

**Table 1:** *Sabellaria spinulosa* reef structure matrix modified from elevation and percentage cover categories proposed by Gubbay (2007)

Reef structure matrix			Elevation (cm)			
			<2	2–5	5–10	>10
			Not a reef	Low	Medium	High
% Cover	<10%	Not a reef	Not a reef	Not a reef	Not a reef	Not a reef
	10–20%	Low	Not a reef	Low	Low	Low
	20–30%	Medium	Not a reef	Low	Medium	Medium
	>30%	High	Not a reef	Low	Medium	High

*Note*

Combination of scores to produce relative scores of “reef status” was based on expert judgment (Fugro, personal communication).

#### 2.3.2 Reef patchiness

Here we define “true patchiness” as:

a value to represent the propensity of *S. spinulosa* reef to be clustered together rather than to grow uniformly and randomly everywhere.

Applying this definition, the size of each patch observed in a video tow can be calculated by creating a presence variable (defined to be: 0 if coverage = 0 and 1 if coverage > 0). A patch is defined as a continuous sequence of values of 1 which is ended by a value of 0. Therefore, if the data for a series of segments comprises the sequence:**1 0 0 1 1 1 0 0 1 1 0**, the resultant patch sizes are calculated as **1**,** 3,** and **2**.

Missing values (e.g., where the seabed is obscured) are excluded, resulting in occasional gaps in the data. For example, where a missing value is * and a series of segment values comprises the sequence shown below: 0 1 * 1 1 0 then the resultant patchiness score would indicate a single patch of **3**.

The definition of patchiness is dependent on the duration of the segments and the mean patchiness statistic used, which for the study reported here is based on 5‐s segments. There may, therefore, be gaps in the reef observations within a segment. The shorter the segment used, the finer the measurement of patchiness because the likelihood of encountering gaps is reduced.

The value of mean patchiness per video tow is determined by the number of segments within which reef is observed (i.e., on reef density). If reef is observed in numerous neighboring segments, the mean patchiness will be higher than if there were fewer neighboring segments with reef present.

To standardize patchiness measurements between reefs, the statistic provided below was calculated:

$$K=p_{o}/p_{r}$$

where *p*_o_ is the mean patch size observed and *p*_r_ is the mean patch size if the presence of reef observations in the data string were random.

The value of *p*_r_ was determined by randomizing the data 1,000 times, calculating the mean patch size each time and then calculating the mean of these 1,000 values. Values of *K* greater than 1 indicate patchiness.

Computation of *K* also allows a *p*‐value to be calculated to test the null hypothesis that the segments where by *S. spinulosa* reef was observed are random. The *p*‐value is calculated from the proportion of times that the mean patch size under randomization is greater than the observed value (Manly, 1998).

#### 2.3.3 Mapping of spatial extent

Sidescan sonar data were accessed using the arcgis 10.1 software package for visualization and interpretation in order to determine the spatial extent of *S. spinulosa* reef. Individual lines were not mosaiced to avoid data loss that can occur during processing. Lines were therefore individually interrogated for reef presence, at a data resolution of 0.3 m. Data collected from each study area (Figure 2) were visually assessed to identify a consistent acoustic signature for *S. spinulosa* reef and to allow polygons indicating the distribution and spatial extent of the reef to be delineated. Acoustic data interrogation was informed by the results of the analyses of accompanying seabed imagery data. Locations where *S. spinulosa* reef was identified from the video transect data were then targeted for further interpretation of potentially indicative acoustic signatures.

Automatic segmentation and classification rely on data values being representative of habitat types to enable effective clustering or object creation. This was not possible using the sidescan sonar data collected here, where raster values varied with distances from the transducers. Alternatively, visual interpretation of sidescan sonar data allows expert judgment to be applied to the habitat delineation process, and as such was more appropriate than automated methods to mitigate the effect of varying raster values caused by the nadir and distance from transducers. The mapped spatial extent of *S. spinulosa* reef therefore represents a combination of observed reef from video analysis and potential reef from sidescan sonar interpretation (i.e., where both acoustic signature and seabed imagery support reef presence).

## 3 RESULTS

### 3.1 Reef status assessment (including patchiness)

Analyses of video using the Cefas standard broad‐scale habitat methodology resulted in videos segmented into large areas where reef was observed and where reef was absent. The percentage cover and elevation of the reef observed was then estimated for each video segment where it was present. Fourteen videos were determined as crossing more than one habitat and were therefore split into segments (from 2 to 8). For example, video tow A68 was determined as having two distinct habitats (segments); Dense cobbles and pebbles with extensive *Sabellaria* reef representing the first habitat (95% of the video) with cobbles and pebbles on fine rippled sand representing the second habitat. Reef elevation and percentage cover were both assessed as high. In contrast, video tow A71 was segmented into 8 sections due to a high level of reef fragmentation. Reefiness was assessed as low‐medium and varied between segments.

Analyses of video transect data, using the 5‐s assessment method, and subsequent analyses of reef patchiness revealed that the best examples of *S. spinulosa* reef occurred in study area A (Figure 1). High values of reef status were observed infrequently across the site. The video transects showing the most spatially extensive areas of reef (A68) were mainly (~66%) assigned Low values of reef status (Table 2).

**Table 2:** Summary of values of *Sabellaria spinulosa* reef status (% cover and elevation) and associated patchiness per video transect ordered according to survey stations where the largest patches of reef were observed

Stn	% Of video tow					No. of patches	Ave patch length	Median patch length	Size range of patches	*K p*_o_/*p*_r_
	No reef	Not reef	Low reef	Medium reef	High reef					
A68	21.39	5.28	65.83	7.50	0.00	28	10.07	5	1–41	2.16
A69	42.59	9.60	41.92	5.89	0.00	47	7.28	5	1–25	3.10
A67	44.87	8.97	39.32	6.62	0.21	56	4.54	2	1–26	2.03
A63	65.53	17.96	11.65	3.88	0.97	19	3.74	2	1–14	2.46

*Note*

Average patch length is defined as the mean number of consecutive (excluding missing observations) 5‐s segments that *S. spinulosa* reef was observed. *K* is the test statistic, defined as the mean observed patch size (*p*_o_) divided by the mean patch size if reef occupancy in a segment was random (*p*_r_) Station code (Stn) refers to the area (e.g., A) and tow number (e.g., 68) collected during the survey.

The *K* values reveal that the four stations, containing most reef, showed patchiness, as reflected by the mean patch sizes all being at least double the value that would be achieved if the reef segment occupations was at random. This patchiness was strongly statistically significant at all stations (*p* < 0.001). Station A69 showed the most patchiness, having a *K* value of 3.1.

### 3.2 Mapping of spatial extent

The acoustic signature identified from sidescan sonar, to be coincident with *S. spinulosa* reef presence, varied across the study site. The potential boundaries were delineated for some of these signatures, but represented areas of known and potential *S. spinulosa* reef presence as was identifiable from the areas where sidescan sonar data were available, rather than exclusively delineating Annex I reef extent within the full extent of the NNSBs and SR cSAC/SCI. Within study area A, there was a demonstrably stronger signature associated with *S. spinulosa* reef presence than at any of the other surveyed areas within the SCI. This is potentially due to the relative reflectivity of the *S. spinulosa* reef in relation to the predominant, adjacent substrate types in study area A in contrast to other study areas (i.e., where the reef reflectivity was less distinct from the surrounding substrate).

### 3.3 *Sabellaria spinulosa* reef in study areas A–F

Limited evidence of *S. spinulosa* reef was identified within study areas B, D, and F. As such results from these study areas are not further presented. Data from these locations is reported in Jenkins et al. (2015).

#### 3.3.1 Study area A

**Figure 3:**
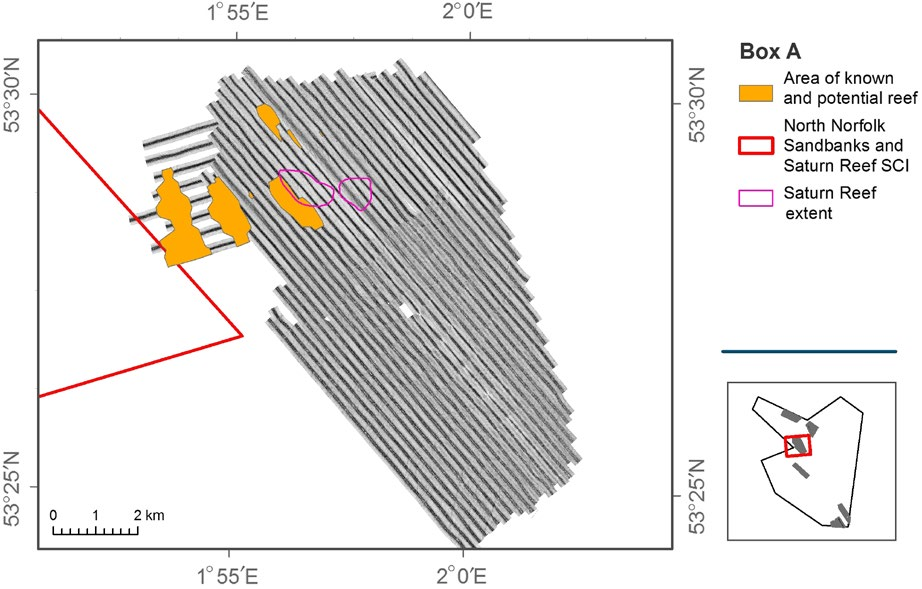
Identified areas of known and potential *Sabellaria spinulosa* reef extent from sidescan sonar in study area A (yellow) and historic location of Saturn Reef (purple)

**Figure 4:**
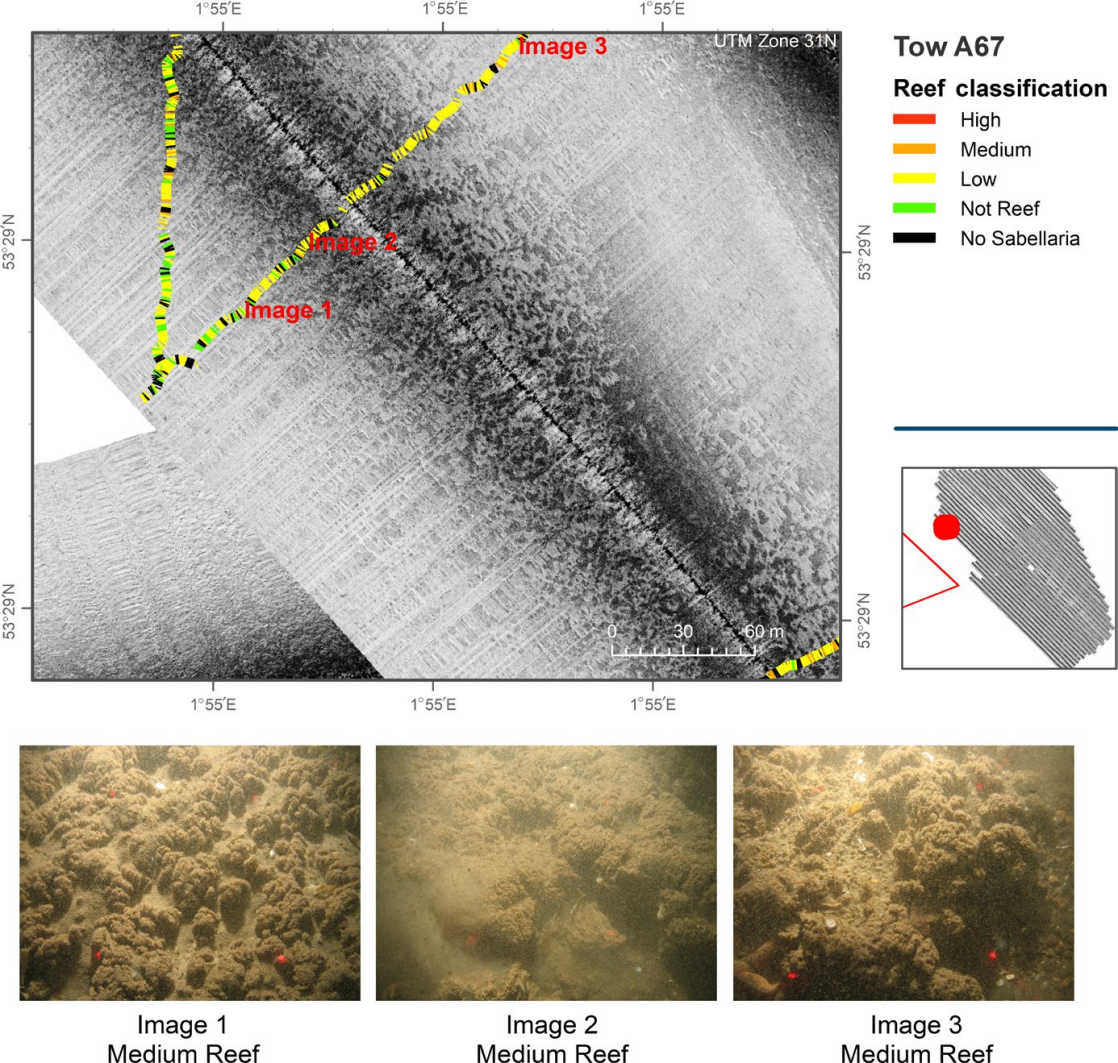
Single sidescan sonar line acquired in study area A (showing the characteristic *Sabellaria spinulosa* reef acoustic signature for this study area) and overlain by results from analyses of seabed imagery data. Video transect broken down into 5‐s intervals and assigned reef classification as detailed in Table 2

**Figure 5:**
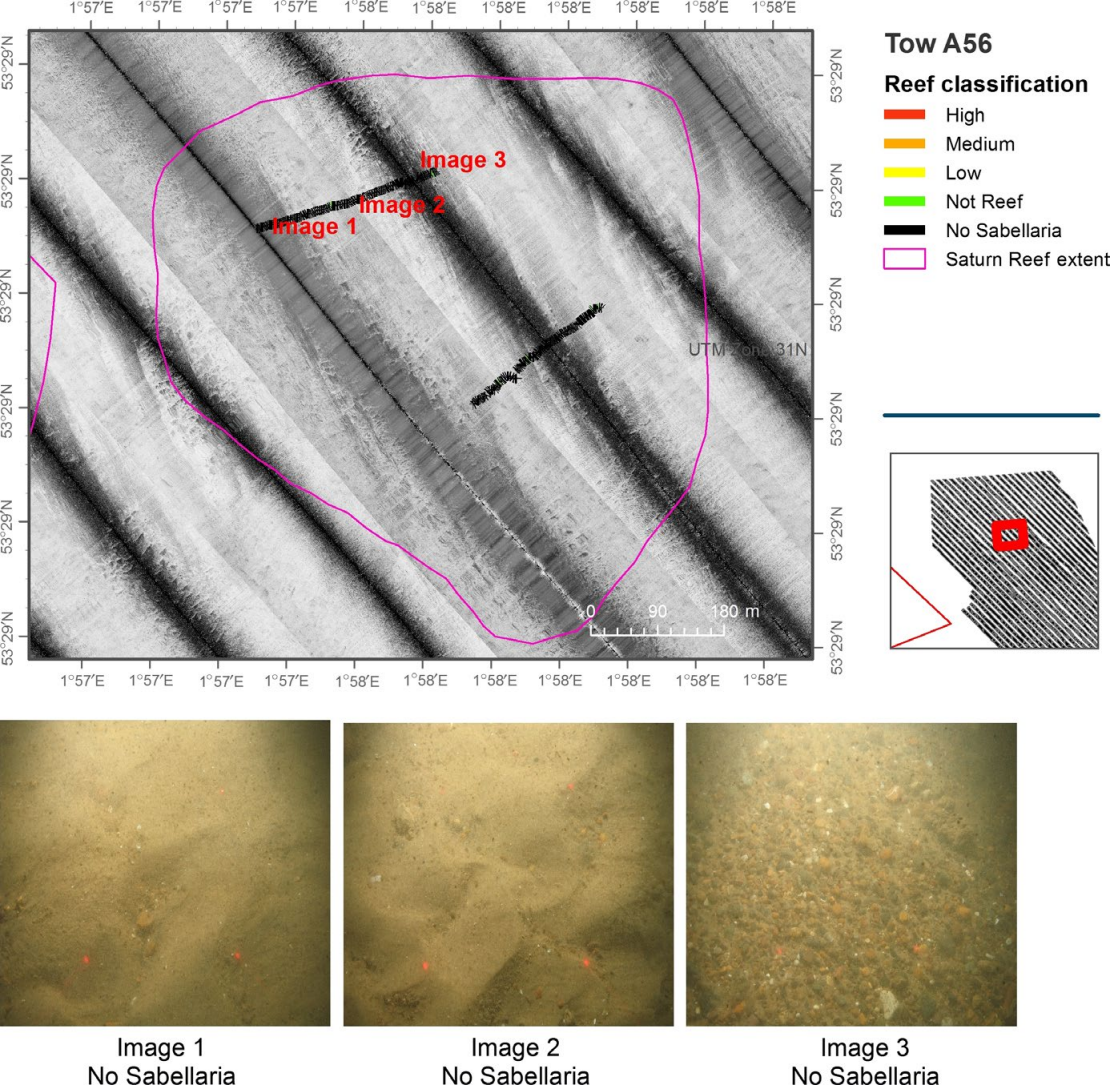
Sidescan sonar and video analysis of the previously reported location of Saturn Reef, overlain by results from analyses of seabed imagery data. Video transects broken down into 5‐s intervals and assigned reef classification as detailed in Table 2

Although areas of *S. spinulosa* were mapped in the study area (Figure 3), and the presence confirmed by video analysis (Figure 4), reefs were delineated at a coarse resolution. Mapping at a finer spatial resolution was not feasible due the nature of the acquired sidescan sonar data. Figure 5 provides the results for video transects and acoustic data acquired directly over the area previously reported to encompass the Saturn Reef feature.

**Figure 6:**
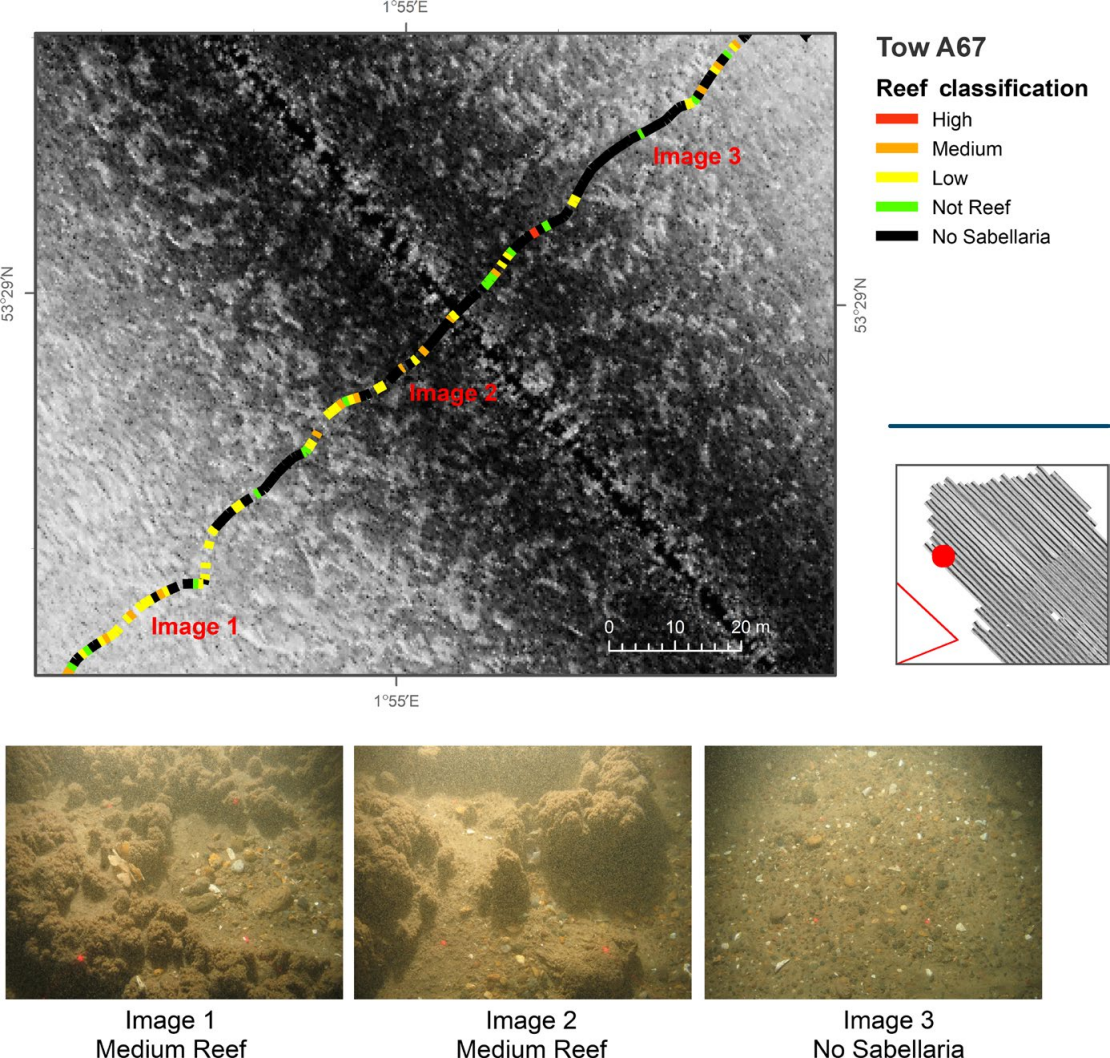
Single sidescan sonar line acquired in study area A and overlain by results from analyses of seabed imagery data. Video transect broken down into 5‐s points and assigned reef classification as detailed in Table 2

Patch sizes of reef observed in study area A varied between 0.004 and 1.5 km^2^. Scores for reef status, assigned as per our reef assessment methodology, included “not reef” to “high reef” (Figures 5 and 6). Areas of known and potential reef were mapped using a precautionary approach to ensure that potential reef areas were considered and included as appropriate. For this reason, spatial extent calculations could represent an over estimation of actual extent *S. spinulosa* reef, according to the accepted definition. It was not possible to extrapolate scores for elevation, percentage cover and spatial extent, from video transects, across mapped areas due to the variable nature and patchiness of the reef structures.

Figure 6 presents a limitation in mapping the spatial extent of *S. spinulosa* reef. The overlaid camera transect demonstrates that video analyses, demarcating areas of reef (see Image 1, Figure 6), intersect regions of both high and low reflectivity derived from the acoustic data. Similarly, areas of “No Sabellaria” (see Image 3, Figure 6) also intersect high and low reflectivity patches.

#### 3.3.2 Study area C

**Figure 7:**
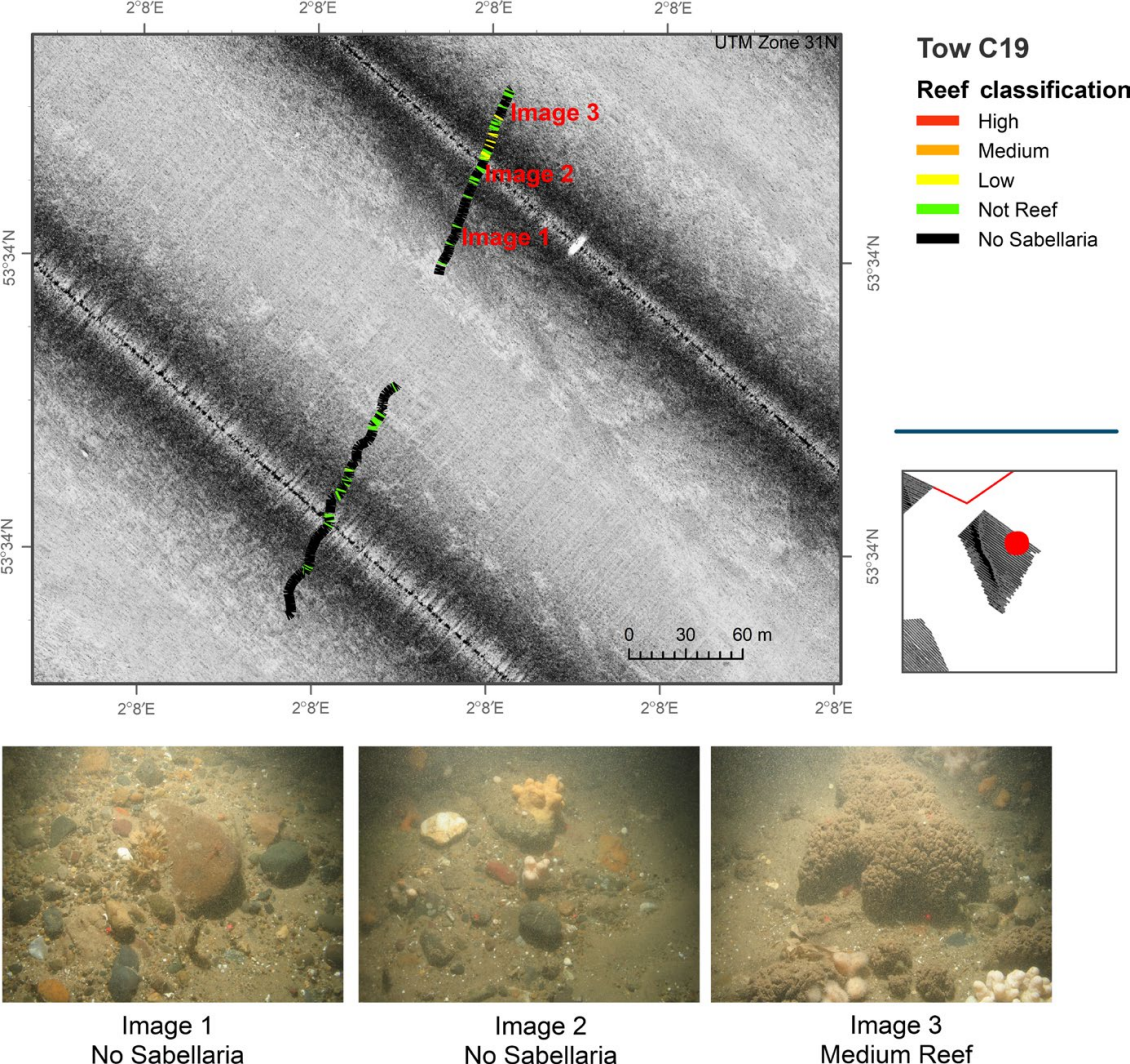
Sidescan sonar data acquired in study area C and overlain by results from analyses of seabed imagery data. Video transect broken down into 5‐s intervals and assigned reef classification as detailed in Table 2

**Figure 8:**
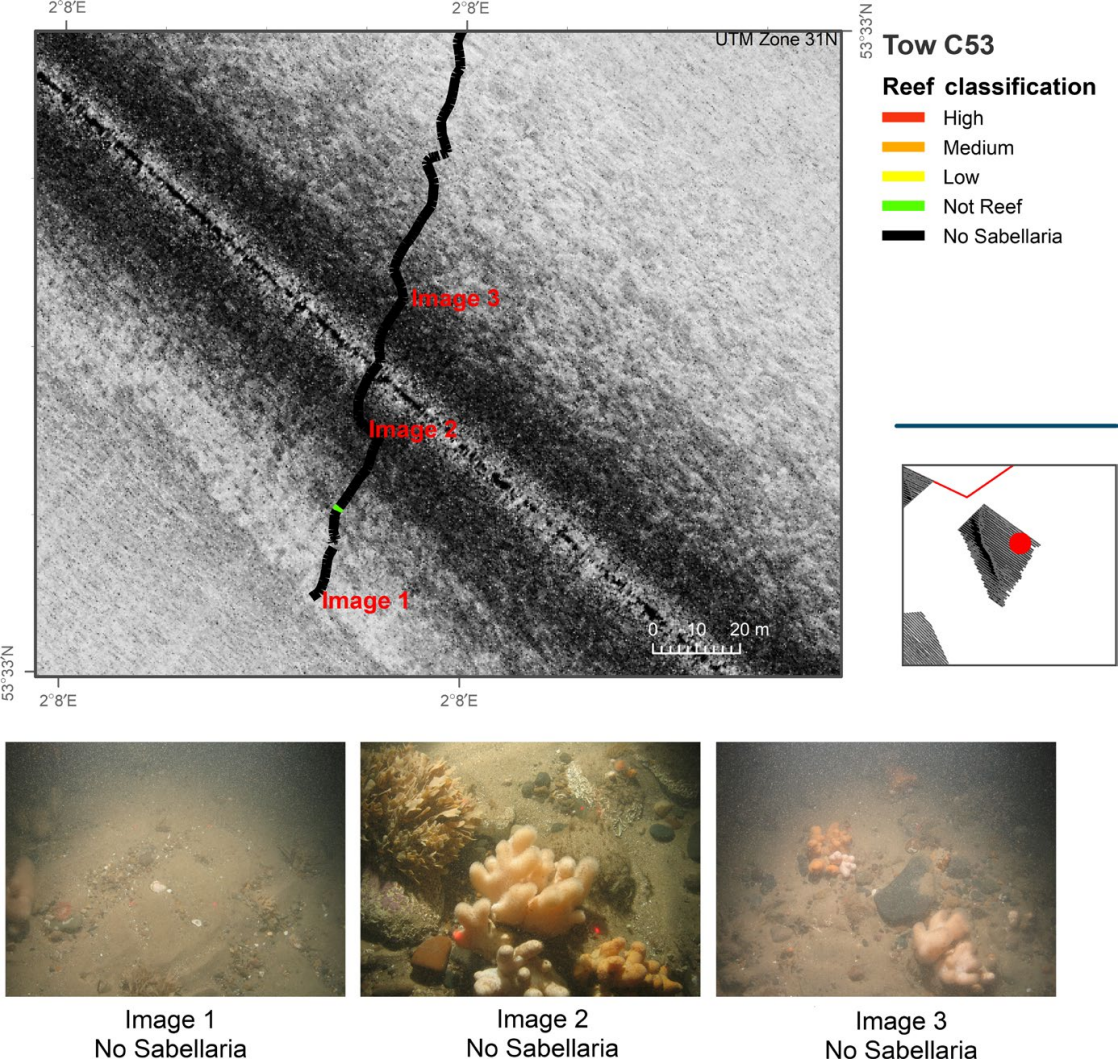
Sidescan sonar data acquired in study area C and overlain with results from analyses of seabed imagery data. Video transect broken down into 5‐s intervals and assigned reef classification as detailed in Table 2

Analyses of video tow data suggest that reef structures assigned a Low reef status are present within this study area. Figure 7 shows an area of *S. spinulosa* reef present with an associated “mottled” signature visible in the sidescan sonar data. This signature was not as clear, and/or pronounced, as that observed in study area A. Further investigation of other tows within study area C (see Figure 8) showed a similar sidescan sonar signature, although no *S. spinulosa* structures were identifiable from the associated video tow data. The predominant substrate identified was a mixture of coarse sediments interspersed with patches of sand. We suggest that the sidescan sonar signature in study area C is a reflection of the harder substrata observed in the video transect data in contrast to adjacent areas of softer sand also present. If this is the case, the acoustic signatures which correlated with *S. spinulosa* presence in ground‐truthing data are most likely a reflection from underlying coarse substrate rather than a replicable reflection generated by reef features.

## 4 DISCUSSION

Using seabed survey data, collected in the southern North Sea in 2013 to assess the presence, spatial extent and status of *S. spinulosa* reef, the study explored methods for: (a) critically assessing survey techniques (especially seabed habitat mapping from remote datasets and condition assessments from video transects), and their limitations; and (b) development of further metrics and repeatable methods for reef assessment.

### 4.1 Assessment of reef status and reef patchiness using seabed imagery data

Drop frame video transects collected across areas of patchy biogenic reef can successfully be used to explore local variability in parameters from which the status of biogenic reef can be derived along with measures of patchiness. Splitting video tows into 5‐s segments for analysis of reef status, using the metrics elevation and percentage cover, gives a local assessment of reef status beyond that achieved from currently proposed methodologies (Coggan et al., 2007; Turner et al., 2016). Aggregating these data across a video transect can be achieved by including a calculation of reef patchiness, along with other summary statistics including median patch length and the size range of patches. The resultant information can then be used to further describe the wider reef status and site condition.

Marine Protected Area site integrity is measured against habitat condition, structure, and function. Biogenic reefs, such as that formed by the Ross Worm *S. spinulosa*, are protected because they provide localized “hotspots” of biodiversity as well as due to observed large‐scale reductions in *Sabellaria* habitat across European waters, especially in the Wadden Sea (OSPAR, 2008). The structural properties of the reefs provide refuge, habitat, and enhance productivity for biodiversity to proliferate beyond what would be occur on sediment or rock alone (Pearce et al., 2011). That being the case, the measures proposed here for reef assessment from drop‐down video transects would support the evidence base underpinning site integrity assessments. Local values for elevation and percentage cover can be estimated accordingly and presented as a habitat map, with summary statistics for reef area patchiness and patch size allowing both spatial and temporal comparisons.

Understanding reef patchiness is a further step forward to better identifying the reef dynamics that underpin the reported biodiversity benefits associated with these reef forming structures. Although more time‐consuming than standard methodologies, utilization of the patchiness measure we present here adds further value and confidence to monitoring and assessing the conservation objectives of those MPAs designated for *S. spinulosa* reef. A potential limitation in making best use of such a patchiness metric is the lack of knowledge relating to the mechanisms by which reef consolidation influences biodiversity. Further work in this area would be highly beneficial.

### 4.2 Mapping the distribution and spatial extent of *Sabellaria spinulosa* using sidescan sonar data

Sidescan sonar signatures that appeared to be associated with patches of *S. spinulosa* identified in seabed imagery data were not consistent across the NNS and SR SCI. A truly unique “mottled” signature, that could be used with sufficient confidence to inform reef delineation, was only found in study area A. Expert judgment is a key component of any such analyses and can introduce a large level of subjectivity when drawing boundaries (Diesing et al., 2014). Due to the nature of the products derived from sidescan sonar data, in this instance, the most appropriate method for assessing reef extent was deemed to be expert manual visual interpretation rather than an automated approach, such as object‐based image analysis (Drăguţ, Tiede, & Levick, 2010). As technologies develop into the future, it is important to continue to explore options for standardizing mapping approaches so that monitoring spatial extent of habitat features as a potential indicator of changes in condition can be assessed with increasing certainty. Sidescan sonar interpretation was hindered in some areas by acquisition at 100% rather than the recommended 200% coverage (due to time constraints), as well as poor weather at the later stages of the survey window. Such limitations should be managed, as much as is possible, for future applications of this methodology to improve replicability of mapping.

From previous work detailed in Limpenny et al. (2010), it is well established that the reliable identification of biogenic reef using remote sensing data challenging and is heavily reliant on a combination of: (a) appropriate technique for acoustic data acquisition, physical nature of the reef structure, density, and reliable georeferencing of ground truthing data and conducive environmental conditions during survey (weather, surrounding habitat, etc.). Pearce et al. (2014) have presented a method for consistent reef mapping using acoustic datasets, whereby newly acquired data from a previously surveyed region have been directly compared. Attempts to apply a similar approach at the NNSB and SR cSAC/SCI were, however, unsuccessful, due to a broadscale habitat backdrop with a similar, and in some regions dominating, acoustic return. Data acquisition is also likely to vary between successive surveys, with weather, line orientation, and processing methods all contributing to potential variability in the final maps being used for comparison, along with the subjective nature of a manual mapping technique which relies on visual interpretation.

Coggan et al. (2007) and Diesing et al. (2014) highlighted the potential sources of error for mapping habitat extent from acoustic sources. Where feasible, data acquisition should utilize the same gear type to ensure consistency of data quality, and postprocessing should similarly follow standardized guidelines to limit inconsistencies.

### 4.3 Implications for *Sabellaria spinulosa* reef monitoring

The effective assessment and monitoring of *S. spinulosa* reef requires that datasets collected through time are accurate and comparable. Measuring reef status and spatial extent is crucial to assessing *S. spinulosa* reef condition. Due to inherent variability within reef areas, it is important for monitoring to establish methodologies that allow consistent and informative metrics to be established, that are representative and comparable between, and within, reef areas.

Although sidescan sonar has been identified as the most appropriate technique for *S. spinulosa* discrimination, as well as other structure forming species (Degraer et al., 2008; Limpenny et al., 2010; Lindenbaum et al., 2008; Wildish, Fader, Lawton, & MacDonald, 1998), the nature of the data products does not currently allow for analyses using machine learning that would help reduce the subjectivity and increase repeatability of the mapping approach.

That being the case, monitoring change in distribution and spatial extent must consider the potential magnitude of error associated with subjective manual habitat mapping and discern whether any measured change in extent is greater than that potential error. Where Pearce et al. (2014) were able to delineate reef relatively clearly within their proposed study area, it has not been feasible within the NNSBs and SR SCI. Future approaches therefore will require careful consideration of the seabed morphology and predominant habitat backdrop as to whether reef extent mapping is achievable or appropriate.

Monitoring of an area, using drop‐down camera system, should not be constrained to static monitoring locations, as repeat tows at a set location cannot guarantee the field of view to be the same as was captured previously. To that end, a random stratified design, where habitat is stratified to areas of reef identified by acoustic techniques, could be utilized. Statistical power analyses can be employed to ensure a sufficient number of video tows are collected to allow significant levels of change to be observed. However, bespoke survey designs should always be employed with careful consideration given to the required outputs.

Survey designs should consider appropriate lengths of individual camera transects for comparability. Where transect lengths cannot be standardized (e.g., when including historical data in analyses), postprocessing should be applied to standardize for the discrepancies. The starting position for each tow should also be considered. For example, where a tow begins a long way from start of a reef area, it will not produce similar summary statistics to transects initiated closer to the reef. For that reason, it is proposed that video analysis, for determination of reef status and patchiness, begins where *S. spinulosa* aggregations are first observed.

As demonstrated by the changes in spatial extent observed over time at the SR location, there is the potential that *S. spinulosa* reef patches are ephemeral. This being the case, there may be high levels of natural variability beyond any anthropogenic impacts, which may hinder our abilities to consistently, and reliably, map *S. spinulosa* reef boundaries. This, in turn, may affect future monitoring and management of these features. In the current absence of appropriate reference areas, we suggest that establishment of those regions with predicted high environmental suitability for *S. spinulosa* reef development may be best mapped at a very coarse scale to reflect *S. spinulosa* reef presence. Combining mapped patches of *S. spinulosa* reef extent to create larger polygons may mitigate for our poor understanding of patch validity and connectivity, as well as for uncertainty in establishing the presence from remote techniques such as sidescan sonar.

Providing the application of this methodology is coupled with a survey design encompassing an appropriate level of stratification and replication we suggest this method will allow for exploration of potential habitat condition variability beyond that shown by reef extent alone. We therefore suggest that MPA site monitoring should harness this technique going forward.

Further work should explore how patchiness of reef areas does or, potentially, does not impact on the functional diversity of the associated fauna and seek to understand the trade‐offs between larger more established reef over lower lying, patchier, reef complexes in the context of a larger, landscape scale perspective. In terrestrial climes, the benefits of coppicing forested areas are well‐known to provide multiple habitat niches that support wider diversity beyond that are associated with an apex community. Marine systems are also known to demonstrate wider benefits from increased habitat diversity, as noted in *Laminaria* forests by Walls et al. (2016). Exploring *S. spinulosa* reef diversity with this in mind may lead to further insight into the function and conservational importance that this biogenic reef habitat is providing.

## 5 CONCLUSIONS

Delineating *S. spinulosa* reef extent was achievable for some areas within the study site, but not for all. The lack of a consistent, and replicable, acoustic signatures synonymous with reef presence across the study site made mapping reef extent at the site scale difficult. Data limitations associated with 100% coverage, rather than the recommended 200% sidescan coverage, as well as poor weather conditions at later stages of the survey further limited data interpretation. Where a potential *S. spinulosa* reef signature was identified, using acoustic techniques, it was possible to combine this with the methodology outlined above to assess reef status along a video transect. The results could then be used to calculate summary statistics, which could be compared between locations and over time. Where applicable, this methodology can therefore be used for the future assessment and monitoring of reef feature at NNSBs and SR SCI. Furthermore, although this was not a primary aim of the study presented here, the novel method for calculating tow patchiness developed in support of the present study delivers a measure of the potential consolidation of a given reef area and may be used to determine the direction of change in reef structure and status over time. The application of this methodology could benefit wider assessments of similar threated or declining habitats such as intertidal *Mytilus edulis* beds on mixed and sandy sediments, Maerl beds, *Modiolus modiolus* beds, *Ostrea edulis* beds, *Zostera* beds, and deep sea sponge aggregations.

## CONFLICT OF INTEREST

None declared.

## AUTHOR CONTRIBUTIONS

All authors were instrumental in conceiving the ideas and designed methodology; C. Jenkins and J. O'Connor collected the data; C. Jenkins, J. Eggleton, and J. Barry analyzed the data; C. Jenkins led the writing of the manuscript. All authors contributed critically to the drafts and gave final approval for publication.

## DATA ACCESSIBILITY

All data within this manuscript have been acquired using public funding and are therefore publically available for request and download. Requests can be made to the authors or downloaded through the MEDIN data portal; http://portal.oceannet.org/portal/start.php.

## Endnotes

1
https://qsr2010.ospar.org/media/assessments/Species/p0010_supplements/CH10_04_Sabellaria_spinulosa.pdf.

2
https://data.gov.uk/data/contracts-finder-archive/download/686209/66dd2669-3904-4804-9861-c464a8fa44f8.
